# Bioinspired Sandcastle Worm-Derived Peptide-Based Hybrid Hydrogel for Promoting the Formation of Liver Spheroids

**DOI:** 10.3390/gels8030149

**Published:** 2022-02-27

**Authors:** Yu-Hsu Chen, Yuan-Hao Ku, Kuo-Cheng Wang, Hung-Chi Chiang, Yu-Pao Hsu, Ming-Te Cheng, Ching-Shuen Wang, Yinshen Wee

**Affiliations:** 1 Department of Orthopedics, Taoyuan General Hospital, Ministry of Health and Welfare, Taoyuan City 330, Taiwan; magister.yuhsu@gmail.com (Y.-H.C.); dryphsu@yahoo.com.tw (Y.-P.H.); cheng1212@mail.tygh.gov.tw (M.-T.C.); 2 Department of General & MIS Surgery, Taoyuan General Hospital, Ministry of Health and Welfare, Taoyuan City 330, Taiwan; gh10161016@gmail.com; 3 Department of Emergency Medicine, Taoyuan General Hospital, Ministry of Health and Welfare, Taoyuan City 330, Taiwan; wkuocheng@gmail.com; 4 Department of Internal Medicine, Division of Neurology Medicine, Taoyuan General Hospital, Ministry of Health and Welfare, Taoyuan City 330, Taiwan; flair1130@gmail.com; 5 School of Dentistry, College of Oral Medicine, Taipei Medical University, Taipei 110, Taiwan; chingshuenwang@tmu.edu.tw; 6 Department of Pathology, University of Utah, Salt Lake City, UT 84112, USA

**Keywords:** hepatic spheroids, peptide-based hydrogel, sandcastle worm adhesive proteins, RGD hydrogel, hepatic differentiation, hybrid hydrogel, 3D culture, improved hepatic function, liver regeneration

## Abstract

The generation of hepatic spheroids is beneficial for a variety of potential applications, including drug development, disease modeling, transplantation, and regenerative medicine. Natural hydrogels are obtained from tissues and have been widely used to promote the growth, differentiation, and retention of specific functionalities of hepatocytes. However, relying on natural hydrogels for the generation of hepatic spheroids, which have batch to batch variations, may in turn limit the previously mentioned potential applications. For this reason, we researched a way to establish a three-dimensional (3D) culture system that more closely mimics the interaction between hepatocytes and their surrounding microenvironments, thereby potentially offering a more promising and suitable system for drug development, disease modeling, transplantation, and regenerative medicine. Here, we developed self-assembling and bioactive hybrid hydrogels to support the generation and growth of hepatic spheroids. Our hybrid hydrogels (PC4/Cultrex) inspired by the sandcastle worm, an Arg-Gly-Asp (RGD) cell adhesion sequence, and bioactive molecules derived from Cultrex BME (Basement Membrane Extract). By performing optimizations to the design, the PC4/Cultrex hybrid hydrogels can enhance HepG2 cells to form spheroids and express their molecular signatures (e.g., *Cyp3A4*, *Cyp7a1*, *A1at*, *Afp*, *Ck7*, *Ck1*, and *E-cad*). Our study demonstrated that this hybrid hydrogel system offers potential advantages for hepatocytes in proliferating, differentiating, and self-organizing to form hepatic spheroids in a more controllable and reproducible manner. In addition, it is a versatile and cost-effective method for 3D tissue cultures in mass quantities. Importantly, we demonstrate that it is feasible to adapt a bioinspired approach to design biomaterials for 3D culture systems, which accelerates the design of novel peptide structures and broadens our research choices on peptide-based hydrogels.

## 1. Introduction

Modeling physiologically relevant events using a traditional two-dimensional (2D) monolayer culture system for drug development, disease modeling, transplantation, and regenerative medicine can be challenging for hepatocytes [1,2,3]. Therefore, culturing cells in 3D culture systems has been developed to enhance the physiological relevance of cell culture systems. It has been demonstrated that primary rat hepatocytes exhibit cell polarity and phenotype reconstruction that resemble native liver tissue when cultured in an extracellular matrix (ECM)-based 3D hydrogel system [4,5]. Additionally, primary hepatocytes that were grown in a 3D culture system have been shown to exhibit increased hepatic functionality and survival capacity [6,7,8]. In general, 3D-cultured hepatic spheroids showed better hepatic gene expression and functionality than traditional 2D culture, suggesting that 3D cultures may provide a more favorable microenvironment for cell regeneration [1,5,7].

ECM-based hydrogels, such as Matrigel^TM^ (Corning, NY, USA), are natural hydrogels obtained from tissues [9,10]. Despite yielding encouraging results as scaffolds for 3D cultures, natural hydrogels may exhibit some batch-to-batch variations that limit their future applications [11,12,13]. Such an issue has become one of the driving forces for improving the performance of natural hydrogels as scaffolds for 3D cultures. For this reason, hybrid hydrogels are of particular interest due to having the advantages of both natural and synthetic hydrogels, which could allow in vitro cell culture in a more controllable and reproducible manner [14,15,16,17,18].

Inspired by glue proteins pc4 from the sandcastle worm *Phragmatopoma californica* [19,20], we designed hybrid hydrogels consisting of secondary structures such as random coils and β-sheet domains to facilitate the growth of hepatic spheroids and improve the reproducibility of spheroid formation. Sandcastle worms secrete glue proteins containing highly repetitive sequences and a relatively simple protein structure that are essential features for the formation of underwater adhesives [19,20,21,22]. For example, the charged amino acid residues (i.e., Tyr and His) in the repetitive motif of pc4 glue proteins interact with other charged glue proteins (i.e., phosphor-Ser) to form an underwater adhesive via the formation of complex coacervates [19,20,23,24,25]. These characteristics make the sandcastle worm glue protein an intriguing model for the development of adhesive materials that can be used in wet environments. In addition, adhesive proteins from the sandcastle worm can fold into complex yet stable molecular architectures in response to various environmental cues (e.g., pH, divalent cations, salt concentrations) [21,23,25,26,27]. Such properties of the adhesion proteins make them attractive targets for the design and development of 3D cell culture scaffolds. Here, we screened the sequences of the potential candidate adhesive proteins from the sandcastle worm and identified those repetitive sequences that would self-assemble into various secondary structures including random coils and β-sheets and form hydrogels. We anticipated that the alternating hydrophilic/charged (i.e., His) and hydrophobic residues (i.e., Leu and Val) of the PC4 peptide (HGVGLHGVGYGLLGRGDS) has the potential to facilitate self-assembly into a stable amphipathic β-sheet structure similar to EAK16 (AEAEARARAEAEARAR) or RADA16 (RADARADARADARADA), which were previously used to form β-sheet hydrogels for cell culture [28,29,30,31,32].

Herein, we report a hybrid hydrogel inspired by sandcastle worm glue protein pc4 combined with the ECM-based natural hydrogel Cultrex, which enhances the physiological relevance for generating functional hepatic spheroids. In this work, we evaluated the influence of hydrogel composition (the proportion of PC4 and Cultrex) on hepatic cells HepG2. To the best of our knowledge, this is the first time that a hybrid hydrogel composed of a peptide domain adapted from sandcastle worm glue protein has been investigated as matrices for hepatic differentiation. First, the cellular toxicity of HepG2 cells in response to hydrogels is characterized, and their differentiation potential is then studied. We concluded that the combination of both materials (PC4 and Cultrex) leads to an environment that promotes a more efficient and robust hepatic differentiation than either one of them used alone. Therefore, our results suggest that these PC4/Cultrex hybrid hydrogels can provide a reliable performance, particularly for 3D culture systems.

## 2. Results and Discussion

### 2.1. Screening of Self-Assembling Peptides Inspired by Sandcastle Worm Adhesion Proteins

**Figure 1:**
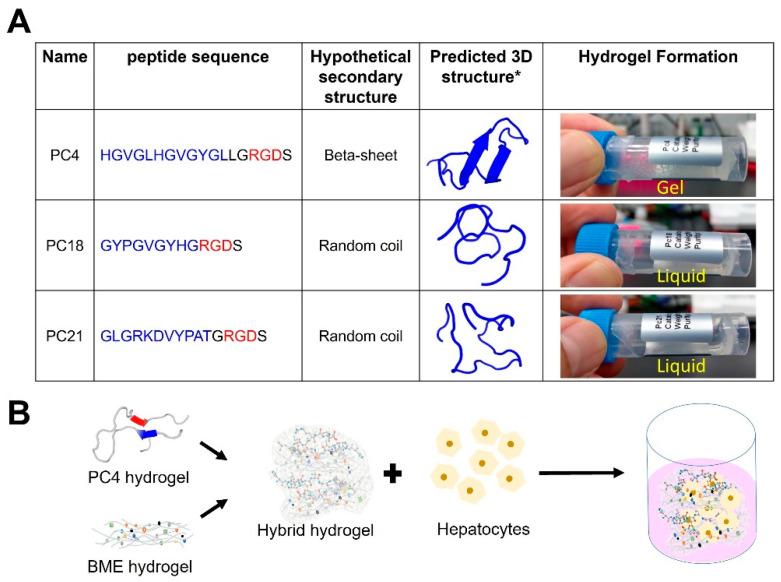
Design and generation of PC4/Cultrex hybrid hydrogel. (**A**) Peptide sequences inspired by the repetitive motif of sandcastle worm adhesive protein (highlighted in blue color). The cell adhesion motif RGD was highlighted in red. * Predicted secondary structure of the peptides using PEP-FOLD 3.5 method. (**B**) The schematic diagram showed the experimental design of hepatocytes encapsulated in the PC4/Cultrex hybrid hydrogel.

In an initial screen, all three tested peptides (PC4, PC18, and PC21) possess different peptide designs and sequence lengths inspired by the repetitive sequence pattern of sandcastle worm adhesion proteins, following a bioactive signaling peptide Arg-Gly-Asp (RGD) cell adhesion sequence to promote the cell-adhesive activity of the hydrogels (Figure 1 and Figure S1A). These pc proteins are identified in the sandcastle worm’s secretory granules and play an important structural role in the formation of the final glue. Pc proteins are basic proteins that all contain at least 10% Gly, His, Lys, and Tyr amino acids with a calculated pI value larger than 8.0 (Figure S1). Their amino acid composition is similar to some natural Gly-rich proteins (i.e., silk protein from Bombyx Mori) that can self-assemble into a β-sheet structure [33,34,35,36]. Therefore, we speculated that our designed PC peptides could also form a β-sheet structure and promote the formation of hydrogel.

PC4 is the only sequence that successfully self-assembled into hydrogels rapidly during our initial screen (Figure 1A). Based on the estimation of PEP-FOLD 3.5 software, PC4 peptide can potentially *form* β-sheets. The potential formation of β-sheet hydrogel found in the PC4 peptide is attributed to the overall hydrophobicity carried by the alternation of Gly, Val, Leu, and His residues presented in the peptides. PC4 is composed of 13 alternating hydrophilic (His and Gly) and hydrophobic (Val, Leu, and Tyr) residues that may drive the formation of β-sheet structures with a hydrophobic face on one side and a hydrophilic face on the other in aqueous media (Figure 1A). A previous report using poly(Gly) incorporated with poly(His) confirmed the formation of a β-sheet hydrogel via hydrophobic aggregation [37]. It is very likely that the high content of Gly presented in the PC4 peptide may also be the driving force of β-sheet formation due to the increased hydrophobicity. Previous findings also demonstrated Gly-rich domains in some natural proteins that self-assemble into a stiff β-sheet structure. For example, silk proteins from Bombyx Mori and suckerins from squid sucker ring teeth are two natural proteins with a high percentage of Gly residues [33,34,35,36,38,39,40,41]. The Gly-rich peptides derived from these natural proteins self-assemble and form fiber-like structures attributed to extended β-sheet formation via hydrophobic aggregations.

PC18 peptide is also a Gly-rich peptide. However, our results show that PC18 with the sequence Ac-(GYPGVGYHGRGDS)-NH2 could be solubilized in aqueous media, but no self-assembling gel formation was observed (Figure 1A). We have another peptide, which is also a Gly-rich peptide with the sequence Ac-(GLGRKDVYPATGRGDS)-NH2 (PC21, supplemental). Again, this peptide could be solubilized in aqueous media, but no self-assembling gel formation was observed. We anticipate that the presence of proline residue in PC18 and PC21 could destabilize β-sheet formation due to the bulky ring structure. These bulky ring structures interfere with the formation of hydrogen bonds, which are critical factors in β-sheet formation [42,43,44].

**Figure 2:**
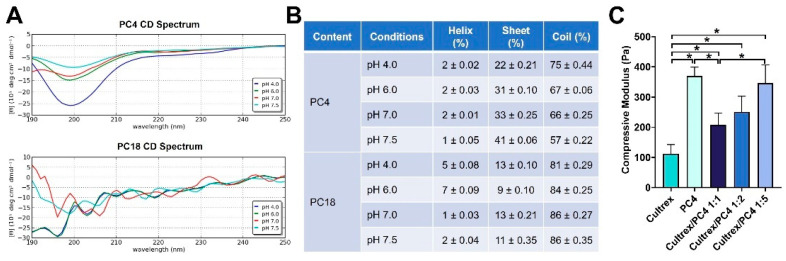
Characterization of PC peptides. (**A**) Circular Dichroism (CD) spectra of 50 µM PC4 and PC18 in 50 mM phosphate buffer at pH 4.0 to 7.5. The peptide was dissolved at room temperature and spectra were recorded at different pH conditions. (**B**) Determination of secondary structure content of PC peptides using CAPITO online CD spectrum analysis. The values are mean ± SD, calculated from triplicate measurements. (**C**) Compressive moduli of PC hydrogels prepared at different combinations. Data are given as mean ± SD; * *p* < 0.05.

In order to better understand the secondary structure of designed peptides, we performed circular dichroism (CD) in phosphate-based buffer at various pH conditions (pH 4.0–7.5). As shown in Figure 2A, the CD spectrum of PC4 showed one negative band in the far-UV region peaked around 198 nm, indicating a random coil structure [45]. Towards higher pH conditions, the spectrum of PC4 changed to less negative ellipticities at wavelengths below 200 nm, indicating that more β-sheet secondary structures occurred (Figure 2A). The CD spectrum of PC18 also showed one prominent negative band at 198 nm, which is dominated by a random coil-like structure as compared to PC4. There are no observable changes in the secondary structure when pH increased in PC18 (Figure 2A).

The quantitative estimation of the relative percentages of α-helical, β-sheet and random coil secondary structure of peptides at different pH conditions were analyzed with CAPITO server-based analysis. As demonstrated in Figure 2B, PC4 peptide at pH 4.0 has a high content of random coil (75%) followed by β-sheet (22%) and helix (2%). When pH increased to 7.5, the content of β-sheet significantly increased to 41% and the content of random coil decreased to 57%, demonstrating the conformation transition from random coil to β-sheet. In contrast, there are no observable changes of secondary structure contents (i.e., helix, sheet and coil) in PC18 at different pH conditions (Figure 2B).

The link between the sequences of amino acids and their ability to form a hydrogel is unclear. Based on the results of PC18 and PC21 peptides, which failed to form hydrogels despite having a high percentage of Gly residues, predicting whether a peptide sequence can form a hydrogel remains challenging. Fortunately, taking inspiration from the sandcastle worm accelerates the design of novel peptide structures for a 3D culture system and broadening our research choices on peptide-based hydrogels. The advantages of using synthetic peptides for hydrogel production can help control aggregation, such as modularity, solubility, and parallel alignment, which may be difficult to achieve with naturally derived peptides and proteins [16,30,39,46,47]. For example, RADA16 has been shown to form a β-sheet hydrogel and has been employed in tissue scaffolding [28,29,46]. However, the presence of carboxylic groups leads to an overall low pH that is harmful to cells [48,49]. Therefore, the use of non-acidic amino acid residues, including alanine, leucine, and valine, can be incorporated into the self-assembled hydrogel system to avoid cell damage. Our designed PC4 comprises non-acidic amino acids (His, Gly, Val, Leu, and Tyr), which provide beneficial effects in cell culture.

### 2.2. Mechanical Strength Evaluation of Self-Assembling Peptides

The compressive moduli of Cultrex, PC4 and PC4/Cultrex hybrid hydrogels at different combinations were shown in Figure 2C. These results suggested that Cultrex BME hydrogel is a soft hydrogel with compressive modulus in the range of 90–100 Pa, whereas PC4 hydrogel is stiffer than Cultrex with compressive modulus around 350–400 Pa. The compressive moduli of PC4/Cultrex hybrid hydrogels at various combinations were found to be in between those of Cultrex and PC4 hydrogels, with an increase in compressive modulus when the concentration of PC4 peptide was increased (Figure 2C).

The formation of liver spheroids depends on environmental cues such as biochemical properties of substrate and stiffness of hydrogels [50,51,52]. These two factors have been reported as equally important for the formation of spheroids. It is generally recognized that hepatocytes cultured in soft Matrigel (<200 Pa) formed spheroids, whereas on stiffer materials or 2D plastic, they do not aggregate and form spheroids effectively [50,51,52,53,54]. The mechanical strength of our 1:1 hydrogel is around 200 Pa. According to our results, cell growth and spheroids formation are more efficient at 1:1 ratio as compared to other culture conditions. These results support the finding of previous studies showing that stiffness of hydrogel is an important factor in determining the size and functionality of hepatic spheroids.

### 2.3. PC4/Cultrex Hybrid Hydrogels Are Biocompatible

**Figure 3:**
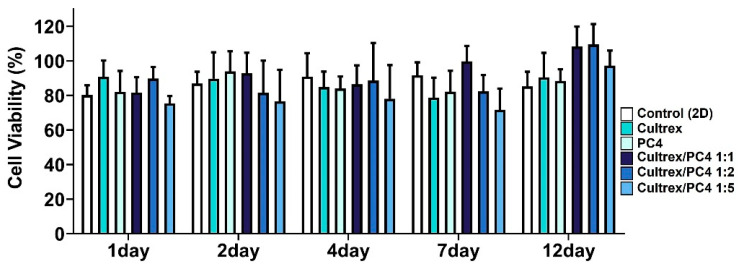
Cell viability in HepG2 cells cultured in different hydrogels. Cells were plated on 96-well plates (1 × 104 cells/well) for various time points with different culture conditions. After incubation for 1 to 12 days, cell viability was measured by MTT assay. No statistical differences were present at all time points in the following groups: 2D, Cultrex, PC4, PC4/Cultrex at 1:1 ratio, PC4/Cultrex at 1:2 ratio, and PC4/Cultrex at 1:5 ratio. Data are expressed as mean ± SD from three independent experiments.

As mentioned previously, the idea underlying the design of the PC4 peptide hydrogel was to incorporate a highly repetitive domain adapted from pc4 adhesive protein in the sandcastle worm (Figure S1A). The results showed that PC4 spontaneously formed a hydrogel in a normal saline buffer containing 150 mM salt at pH 7.4 (Figure 1). To create a hybrid hydrogel, we mixed a peptide hydrogel (PC4) with an ECM hydrogel (Cultrex) at various ratios (1:1, 1:2, and 1:5), and these mixtures were used to encapsulate HepG2 cells (Figure 1B). To confirm the capability of the hybrid hydrogel to support the viability of HepG2 when encapsulated in the 3D hybrid hydrogel. Cell viability was therefore assessed using a MTT assay over a period of 12 days of culture. It is clear from the results shown in Figure 3 that HepG2 remained viable over 12 days of culture within the hybrid gel under all conditions.

**Figure 4:**
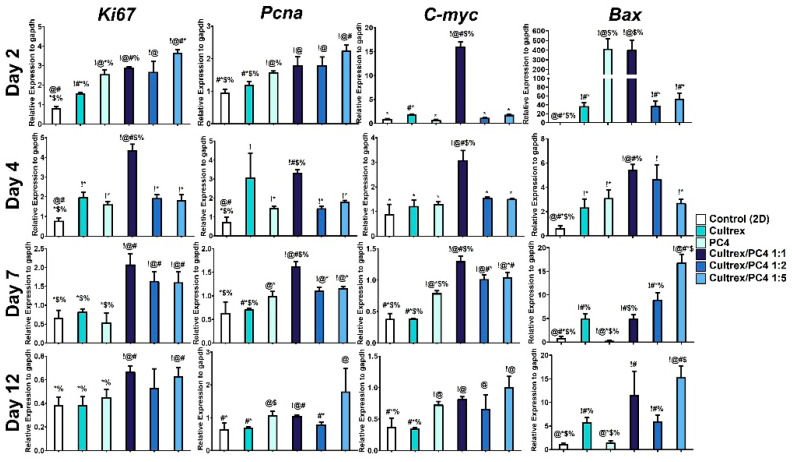
Gene expression of cell proliferation markers (*Ki-67, Pcna, c-myc*, and *Bax*). After HepG2 cells were cultured in different conditions from day 2 to 12, the mRNA expression profile was determined by qPCR. ! indicates groups compared with control (2D). @ indicates groups compared with Cultrex. # Indicates groups compared with PC4. * indicates groups compared with Cultrex/PC4 1:1. $ indicates groups compared with Cultrex/PC4 1:2. % indicates groups compared with Cultrex/PC4 1:5. The results from the three independent experiments are presented as the mean ± standard deviation. !, @, #, *, $, % *p* < 0.05.

To further explore the proliferative activity of HepG2 cells in hydrogels, we determined the cell proliferation markers *Pcna, Ki-67, c-myc*, and *Bax* at the mRNA level. As shown in Figure 4, there was a significant increase in the mRNA expression of *Ki-67, Pcna, c-myc*, and *Bax* in cells cultured in 3D hydrogels (i.e., Cultrex, PC4, and PC4/Cultrex), whereas the most significant upregulation of these genes was observed in cells cultured with 1:1 ratio of PC4/Cultrex at all measured time points (day 1–12). Our results are similar to a previous study showing that a hybrid hydrogel containing GRGDS peptide and polyisociano peptide polymer is capable of inducing cell proliferation, migration, and differentiation of fibroblasts [55]. Moreover, another hybrid scaffold, which includes peptide- and polymer-based hydrogels, such as RADA and PEG gels, was shown to maintain the viability of rat neural stem cells and hMSCs [56,57].

### 2.4. PC4/Cultrex Hybrid Hydrogels Significantly Promote Hepatic Spheroid Formation

**Figure 5:**
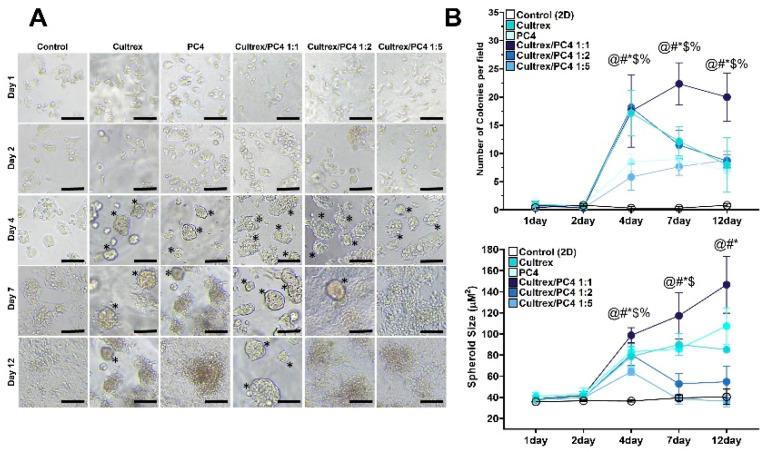
Effect of hydrogels on the growth and size distribution of HepG2 spheroids. Cells were grown in different hydrogels or control (2D) condition. The number and size distribution were determined at day each time point from day 1 to 12. (**A**) Bright field images to monitor the growth of HepG2 spheroids in size and number. Black asterisks indicate cell aggregates of diameter larger than 35 μm that were considered spheroids. Scale bars = 100 μm. (**B**) Comparison of numbers and mean diameter of HepG2 spheroids over 12 days. @ indicates Cultrex compared with control (2D). # indicates PC4 compared with control (2D). * indicates Cultrex/PC4 1:1 compared with control (2D). $ indicates Cultrex/PC4 1:2 compared with control (2D). % indicates Cultrex/PC4 1:5 compared with control (2D). Data are expressed as the mean ± SD of 10 images in each condition. @, #, *, $, % *p* < 0.05.

The light microscopy images showed that HepG2 cells rapidly proliferated and converted into spheroids in most 3D conditions (i.e., Cultrex, PC4, and PC4/Cultrex) than those grown as a 2D monolayer (Figure 5A). Regarding the morphological changes in these 3D conditions, we found that cells grown in BME-based Cultrex formed spheroids at day 4 and disaggregated at day 12. Similarly, PC4 peptide hydrogel promoted the formation of spheroids at day 4 and gradually disaggregated at day 7 and 12 (Figure 5A). Cells continuously formed and maintained in spheroids were only found in hybrid hydrogel at 1:1 ratio of PC4/Cultrex (Figure 5A, marked with asterisks). In contrast, hybrid hydrogel containing a higher ratio of Pc4 (i.e., PC4/Cultrex at 1:2 and 1:5), although promoting cell growth, did not promote the formation of spheroids (Figure 5A). When further examining the growth of spheroids in 3D conditions, we found that the number and size of spheroids were significantly higher in the hybrid hydrogel at 1:1 ratio of PC4/Cultrex as compared to other 3D conditions at day 4, 7, and 12 (Figure 5B). This result indicates that the microenvironment created by hybrid hydrogel at 1:1 ratio of PC4/Cultrex is favorable for the proliferation and differentiation of HepG2 cells, which in turn lays a foundation for further development of liver cell functions.

Previous studies have demonstrated that necrosis likely occurs in spheroids of diameter larger than 260 μm due to hypoxia stress [58,59]. In our culture conditions, the diameters of HepG2 spheroids were less than 200 μm (Figure 5A). Moreover, cell viability across all culture conditions remains unchanged over time (Figure 3). Therefore, it is less likely that necrotic cell death occurred in our system.

### 2.5. PC4/Cultrex Hybrid Hydrogels Significantly Enhanced the Liver-like Functionality of Hepatic Spheroids

**Figure 6:**
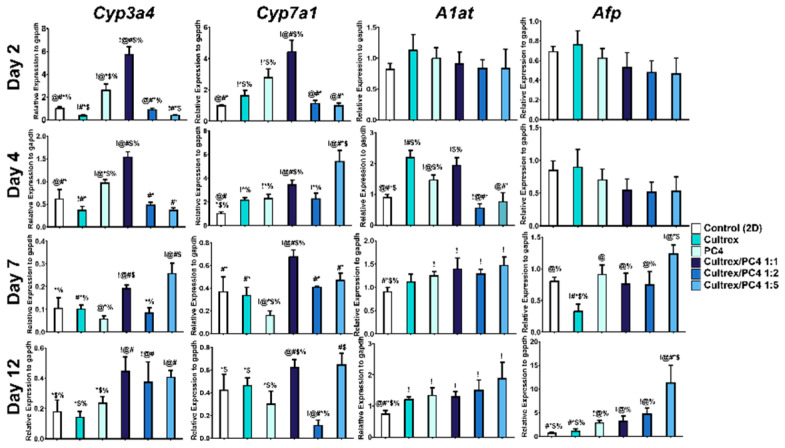
Gene expression of hepatic functional markers (*Cyp3a4, Cyp7a1, A1at*, and *Afp*). After HepG2 cells were cultured in different conditions from day 2 to 12, the mRNA expression profile was determined by qPCR. ! indicates groups compared with control (2D). @ indicates groups compared with Cultrex. # indicates groups compared with PC4. * indicates groups compared with Cultrex/PC4 1:1. $ indicates groups compared with Cultrex/PC4 1:2. % indicates groups compared with Cultrex/PC4 1:5. The results from the three independent experiments are presented as the mean ± standard deviation. !, @, #, *, $, % *p* < 0.05.

Spheroids of HepG2 cells have been shown to exhibit enhanced liver-like functionality compared to 2D cultures by the upregulation of genes involved in liver metabolism and maturation [60,61], making it a more realistic liver model. In general, HepG2 spheroids are described as 3D cultures with a high activity of liver-specific functions, such as alpha-fetoprotein (*Afp*), CYP enzymes, alpha-1 antitrypsin (*A1at*), cytokeratins, and E-cadherins expression when compared to 2D cultures [62,63,64]. In order to compare the metabolic status between the 2D and 3D HepG2 cultures, the gene expression of these liver-like functionalities was measured. First, we analyzed genes that are involved in liver metabolism, such as *Cyp3a4, Cyp7a1, A1at*, and *Afp*. Compared with cells cultured in 2D (control) and embedded 3D culture, the HepG2 cells cultured in PC4/Cultrex hydrogel at 1:1 ratio showed the highest levels of hepatic gene (*Cyp3a4* and *Cyp7a1*) expression at all time points (day 1–12) (Figure 6). The expression of *A1at* and *Afp* was not significant in all conditions at day 1 and 2. However, *A1at* and *Afp* were upregulated in hybrid hydrogels (ratio 1:1, 1:2, and 1:5) from day 4 to 12, in which ratio 1:5 showed the highest expression (Figure 6). Other 3D culture conditions including Cultrex, PC4, 1:2, and 1:5 hybrid hydrogels showed significant upregulation of genes related to liver metabolism, but the expression levels varied across the different time points. In contrast, 1:1 ratio of PC4/Cultrex showed consistent and significant upregulation of genes that are involved in liver metabolism throughout most of the time points that were measured.

**Figure 7:**
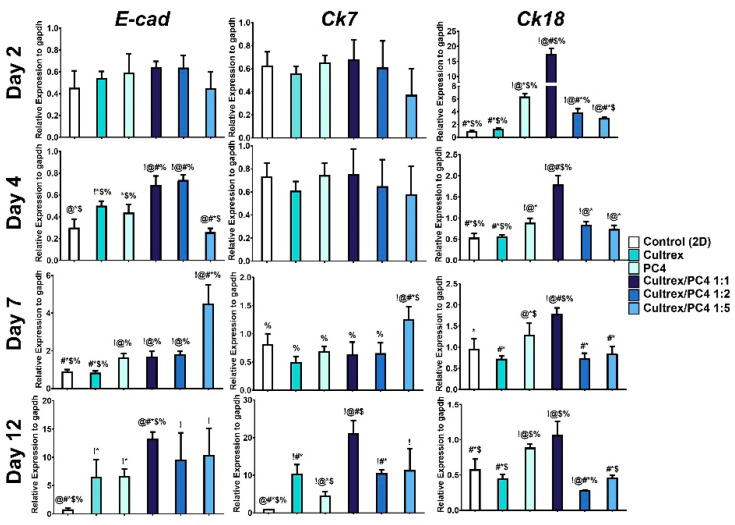
Gene expression of hepatobiliary progenitor markers (*E-cad, Ck7*, and *Ck18*). After HepG2 cells were cultured in different conditions from day 2 to 12, the mRNA expression profile was determined by qPCR. ! indicates groups compared with control (2D). @ indicates groups compared with Cultrex. # indicates groups compared with PC4. * indicates groups compared with Cultrex/PC4 1:1. $ indicates groups compared with Cultrex/PC4 1:2. % indicates groups compared with Cultrex/PC4 1:5. The results from the three independent experiments are presented as the mean ± standard deviation. !, @, #, *, $, % *p* < 0.05.

Gene expression related to liver maturation markers, such as Cytokeratin 7 (*Ck7*), Cytokeratin 18 (*Ck18*), and E-cadherin (*E-cad*) were analyzed. Compared with cells cultured in 2D and 3D culture conditions, the cells cultured in PC4/Cultrex hydrogel at 1:1 ratio showed the highest levels of hepatocyte-associated intermediate filament *Ck18* expression at all time points (day 1–12) (Figure 7). In general, the gene expression profiles of *Ck7, Ck18*, and *E-cad* were significantly higher in the PC4/Cultrex hydrogel compared to other 3D conditions (i.e., Cultrex and PC4) and 2D control (Figure 7). Altogether, our results showed that the hybrid hydrogel at 1:1 ratio of PC4/Cultrex provides a more favorable microenvironment for HepG2 cells to self-organize and differentiate to form functional hepatocytes.

ECM proteins, such as collagen IV, laminin, and fibronectin, not only provide structural support for cells but also play crucial and complex roles during cell signaling [65,66]. Attempts to design/generate the matrices for controlled cell signaling have been more difficult to achieve with in vitro culture systems. Such difficulties can be overcome by using self-assembling peptide hydrogels which are an advanced type of synthetic scaffold, which may integrate functional, mechanical, chemical, and biological cues [47,67]. For example, cell adhesion motifs such as Arg-Gly-Asp (RGD) can be incorporated into self-assembling peptide hydrogels to induce cell attachment and proliferation [67,68]. It has been demonstrated that the incorporation of RGD motifs promotes the formation of spheroids with improved viability in ovarian and liver in vitro models [67,69]. This type of synthetic peptide-based hydrogel not only easily supports 3D cell organization but also introduces biological cues into the scaffolds that mediate cell–cell adhesion and cell–matrix interactions in a user-directed manner.

Studies show an improved function of hepatocytes by the upregulation of genes involved in liver metabolism and maturation (e.g., albumin, *A1at, Cyp3a4, E-cad*, and cytokeratins) when utilizing ECM components (i.e., collagen IV, laminin, and fibronectin) to culture hepatocytes [62,63,64]. Similarly, gene expression related to liver metabolism and maturation was upregulated when PC4/Cultrex hybrid hydrogels were used to culture hepatocytes (Figure 6 and Figure 7). These results indicate that PC4/Cultrex hybrid hydrogels significantly enhanced the liver-like functionality of hepatic spheroids, suggesting PC4/Cultrex hybrid hydrogels serve as a better reservoir for bioactive materials and have a structural support closely resembling that of hepatic native ECM.

In sum, this study demonstrates the successful application of 3D hepatic spheroid generation that is versatile and cost effective. Importantly, we further showed that hydrogels designed by natural inspiration can be used in biomedical applications such as cell culture. There is still much we can learn from the highly repetitive proteins found in nature (i.e., the sandcastle worm), as their naturally occurring tandem repeat proteins are sources of inspiration for designing and modifying more advanced biomimetic materials for liver regeneration.

## 3. Conclusions

In this study, we presented a functionally enhanced 3D in vitro hepatocyte culturing model that may be more likely to reflect true physiological in vivo. In addition, in vitro assays using a 3D culture system can produce functional hepatocytes and provide more accurate data on compound activity, which will contribute to improving the efficiency of drug discovery, reducing drug development costs, and accelerating research on liver regeneration. These benefits will be valuable to drug development, disease modeling, transplantation, and regenerative medicine applications. Overall, we demonstrated that a hybrid hydrogel composed of RGD peptide and ECM components could be employed to synergistically enhance hepatic function as we influence spheroid culture. In particular, taking advantage of a bioinspired approach to designing biomaterials for 3D culture systems, we can not only accelerate the design of novel peptide structures but also broaden our research choices on peptide-based hydrogels.

## 4. Materials and Methods

### 4.1. Peptide Design

The initial screen of potential hydrogel-forming peptides was conducted using amino acid sequences adapted from sandcastle worm’s highly repetitive region of the adhesive protein [19,20] and analyzed them with PEP-FOLD 3.5 online server (https://mobyle.rpbs.univ-paris-diderot.fr/cgi-bin/portal.py#forms::PEP-FOLD3, access on 21 October 2021) via de novo structural reconstructions, to simulate the secondary structures (i.e., random coil or β-sheet). PC4 (Ac-HGVGLHGVGYGLLGRGDS-Am), PC18 (Ac-GYPGVGYHGRGDS-Am) and PC21 (Ac-GLGRKDVYPATGRGDS-Am) peptides were synthesized using standard 9-fluorenylmethoxycarbonyl chemistry for solid-phase peptide synthesis on an Advanced Chemtech Apex 396 multipeptide automated synthesizer. The peptide was then purified with a high-performance liquid chromatograph (HPLC) using a preparative reverse-phase C-18 column and then dialyzed against deionized water to remove salts. The PC4 peptide was then analyzed by electron-spray ionization time of flight (TOF) mass spectrometry on a Bruker microTOF. The results of HPLC and mass spectrum are provided in the Supplementary Information (Figures S2 and S3).

### 4.2. Hydrogel Preparation

PC4, PC18, and PC21 peptides (>95% purity) were used to prepare a peptide solution at 2.5 wt%. The peptide powder was dissolved in 800 µL of double-distilled water (ddH_2_O). To induce the formation of hydrogel, NaOH was used to adjust pH to 7.5 in phosphate-buffered saline. A further incubation at 37 °C for 12–24 h was applied to the hydrogel before finally being cooled at room temperature (RT) to ensure the formation of a homogeneous hydrogel. Cultrex BME hydrogel was prepared according to the manufacturer’s instructions. The hybrid hydrogels were prepared by mixing Cultrex and PC4 with different volume ratios (PC4/Cultrex at 1:1, 1:2, and 1:5, respectively).

### 4.3. Mechanical Characterisation of Hydrogels

Hydrogels were prepared in the 24-well plate with a final volume at 1 mL. 50 mM phosphate buffer was added to rehydrate and stabilize hydrogels for 24 h before testing. Unconfined compression testing with a 5 N load cell was performed using a 5 kN MTS Insight load frame material testing system (MTS Systems). Compression testing was performed at a 2 mm/min strain rate to 80% strain. The modulus was calculated using the slope of the linear portion of the stress–strain curve plot.

### 4.4. Circular Dichroism Spectroscopy (CD)

The CD spectra of hydrogels were recorded on an Aviv Model 410 circular dichroism spectrometer (AVIV Biomedical Inc, Lakewood, NJ, USA) using quartz cuvettes with a path length of 1 mm. Spectra were collected in the 190–250 nm spectral range and averaged over three scans at room temperature. All the scans were carried out at a scan speed of 50 nm/min, with a bandwidth of 1 nm and time-response parameters set to 2 s. A reference spectrum of distilled water was recorded and subtracted from each spectrum. The estimation of the peptide secondary structure was achieved by using CAPITO (https://data.nmr.uni-jena.de/capito/index.php, accessed on 15 February 2022).

### 4.5. In Vitro Cell Culture

Human HepG2 hepatic cells were purchased from the American Type Culture Collection (BCRC, Taiwan). Cells were cultured at 37 °C in 5% CO_2_ in growth medium (Dulbecco’s modified Eagle medium, DMEM, Gibco/BRL, Carlsbad, CA, USA) supplemented with 10% (*v*/*v*) fetal calf serum (FBS, Gibco/BRL), and an antibiotics cocktail. Cells were seeded at equal densities directly into the wells of a standard 6-well plate (Nunc, Roskilde, Denmark). The growth medium was changed every 3 days.

### 4.6. MTT Assay

The number of viable cells was determined using a commercially available colorimetric MTT assay (Promega, Madison, WI, USA) according to the manufacturer’s instructions. In brief, the measurement of cell viability was based on the conversion of tetrazolium salt into a blue formazan product detectable by a spectrophotometer (570 nm). The assay was performed on HepG2 cells cultured on 2D and 3D substrates for various periods (day 1 to 12) under different growth conditions.

### 4.7. Spheroid Size Evaluation

The spheroid size and number were evaluated by measuring the spheroid perimeter using Image J software (NIH, Bethesda, MD, USA). In brief, all images were converted to simplified threshold images under the same converting condition, and the edges of the spheroids were then detected using a selection tool. Diameters of the selected spheroid were measured initially as pixels and converted to micrometers by comparing them to a reference length. Cell aggregates of diameters larger than 35 μm were considered spheroids.

### 4.8. Quantitative Real-Time Polymerase Chain Reaction

HepG2 cells cultured in different conditions at each time point were harvested and lysed with a Trizol reagent (Life Technologies, Carlsbad, CA, USA), followed by RT-qPCR protocol as previously described [70]. The oligonucleotides were synthesized by IDT Co., Ltd. (Coralville, IA, USA). The primers used in PCR are listed in Table S1. Relative fold changes in gene expression were normalized using *Gapdh*, and results were plotted and analyzed using Prism software (GraphPad Software Inc., San Diego, CA, USA).

### 4.9. Statistics

Data were mean ± SD of the results from three or more experiments. *p* < 0.05 were calculated from a two-tailed t-test or a two-way analysis of variance (ANOVA) with Prism (GraphPad Software Inc.) and taken to represent significant differences.

## Data Availability

The data presented in this study are available in this article and in the supplementary material.

## Supplementary Materials

The following supporting information can be downloaded at: https://www.mdpi.com/article/10.3390/gels8030149/s1, Figure S1: Amino acid composition of sandcastle worm’s adhesive proteins; Figure S2: MS Spectrum of PC4 peptide; Figure S3: HPLC report of PC4 peptide; Table S1: Primer sequences used in this study.
