# Supplementary material for: Bioinspired Sandcastle Worm-Derived Peptide-Based Hybrid Hydrogel for Promoting the Formation of Liver Spheroids
Source: Gels. 2022 Feb 27;8(3):149. doi: 10.3390/gels8030149 (PMC8950079; doi:10.3390/gels8030149)
Supplement: Supplementary file 1 [file gels-08-00149-s001.zip › gels-1579894-supplementary.pdf]

gels

MDPI

Supplementary Materials

Bioinspired Sandcastle Worm-Derived Peptide-Based Hybrid Hydrogel for Promoting the Formation of Liver Spheroids

Yu-Hsu Chen [1,†], Yuan-Hao Ku [2,†], Kuo-Cheng Wang [3,†], Hung-Chi Chiang [4], Yu-Pao Hsu [1], Ming-Te Cheng [1], Ching-Shuen Wang [5] and Yinshen Wee [6,*]

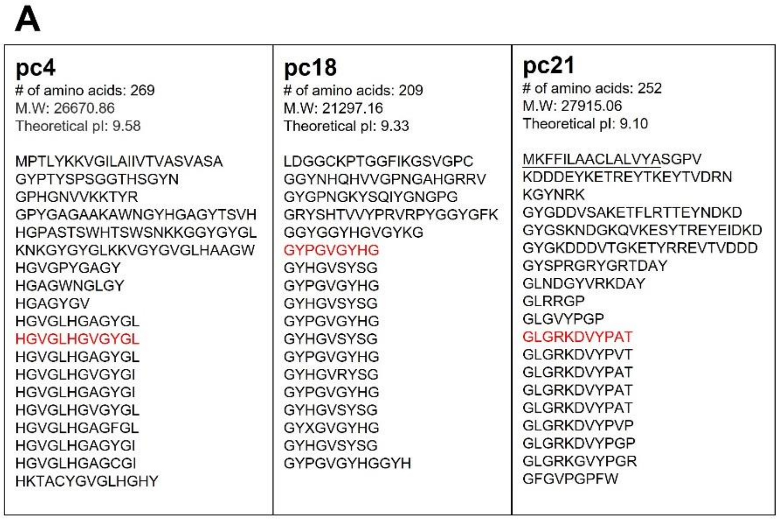

B

Amino acid composition (mol %)

a.a	Pc4	Pc18	Pc21
Ala	8.2	0.5	4.4
Arg	0.4	2.9	8.7
Asn	2.2	1.9	2.0
Asp	0	0.5	10.7
Cys	0.7	1.0	0.4
Gln	0	1.0	0.4
Glu	0	0	4.4
Gly	31.2	35.9	16.7
His	11.5	9.1	0
Ile	2.6	1.0	0.8
Leu	7.8	0.5	6.0
Lys	4.5	2.4	9.1
Met	0.4	0	0.4
Phe	0.4	1.0	2.0
Pro	2.6	6.2	6.7
Ser	3.7	6.7	2.0
Thr	3.3	1.0	6.3
Trp	1.9	0	0.4
Tyr	9.7	18.2	10.3
Val	8.9	10	8.3

Figure S1. Amino acid composition of sandcastle worm's adhesive proteins.

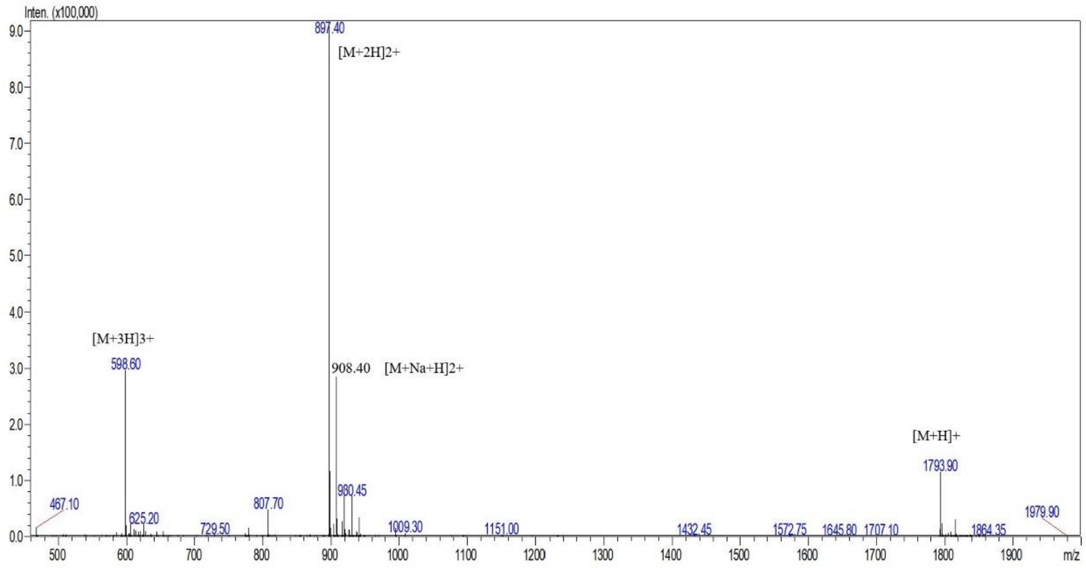

Mw: 1792.94
Probe: ESI
Probe bias: +4.5kv
Nebulizer Gas Floe: 1.5L/min
Detector: 1.2kv
CDL: -20.0v

Figure S2. MS Spectrum of PC4 peptide.

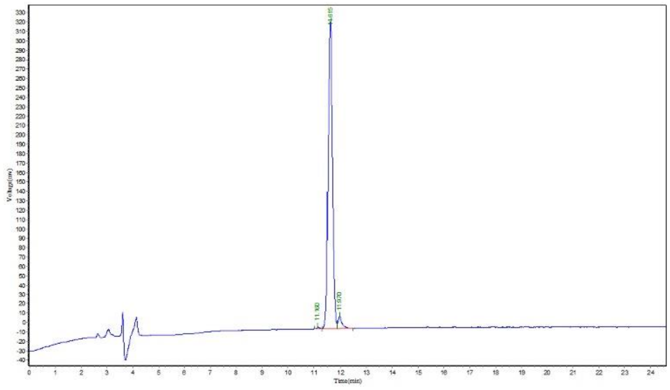

Peak No.	Ret Time	Height	Area	Conc.
1	11.160	1688.932	12554.857	0.3317
2	11.615	326223.031	3636195.250	96.0547
3	11.970	12748.593	136797.188	3.6137
Total				100.00

Column :250*4.6mm,Kromasil-C18-5um
Solvent A:0.1%TFA in 100%water
Solvent B:0.1%TFA in 100%acetonitrile

Gradient :	A	B
0.1min	79%	21%
25.0min	54%	46%
25.1min	0%	100%
30.0min	stop	

Flow rate:1.0ml/min
Wavelength(nm):220
Volume :10ul

Figure S3. HPLC report of PC4 peptide.

Table S1. Primer sequences.

Gene	Forward (5′-3′)	Reverse (5′-3′)
**PCNA**	CCTGCTGGGATATTAGCTCCA	CAGCGGTAGGTGTCGAAGC
**c-Myc**	ATGGCCCATTACAAAGCCG	TTTCTGGAGTAGCAGCTCCTAA
**Ki67**	TTCGCAAGCGCATAACCCA	AACCGTGTCACAGTGCCAAA
**bax**	CCCGAGAGGTCTTTTTCCGAG	CCAGCCCATGATGGTTCTGAT
**CYP3A4**	TTCAACAGATGATCGACTCCCA	TTGTGTCATAGGCAGCAAAAATG
**CK18**	TCGCAAATACTGTGGACAATGC	GCAGTCGTGTGATATTGGTGT
**CYP7A1**	AGAAGCATTGACCCGATGGAT	AGCGGTCTTTGAGTTAGAGGA
**A1AT**	CCTGAAGGCCCCTGATAAGAA	GCTGGACTGATTGAGGGTGC
**CK7**	GTGGCAGCCGAAGTTAGGAAG	GCACTGTGGTGTGATGGGATA
**AFP**	CTTTGGGCTGCTCGCTATGA	GCATGTTGATTTAACAAGCTGCT
**E-cadherin**	CGAGAGCTACACGTTCACGG	GGGTGTCGAGGGAAAAATAGG
